# Numerical study of the risk of thrombosis in the left atrial appendage of chicken wing shape in atrial fibrillation

**DOI:** 10.3389/fcvm.2022.985674

**Published:** 2022-11-25

**Authors:** Jun Yang, Chentao Song, Huirong Ding, Mu Chen, Jian Sun, Xiaohua Liu

**Affiliations:** ^1^School of Energy and Power Engineering, University of Shanghai for Science and Technology, Shanghai, China; ^2^Xinhua Hospital, School of Medicine, Shanghai Jiao Tong University, Shanghai, China; ^3^School of Aeronautics and Astronautics, Shanghai Jiao Tong University, Shanghai, China; ^4^Key Laboratory (Fluid Machinery and Engineering Research Base) of Sichuan Province, Xihua University, Chengdu, China

**Keywords:** atrial fibrillation, computational fluid dynamics, left atrium, left atrial appendage, thrombus

## Abstract

Atrial fibrillation (AF) is a common and life-threatening disease. For the patients with AF, more than 90% of the thrombi are formed in the left atrial appendage (LAA), thrombus dislodgement can cause vascular embolism, making them is becoming a high-risk group for stroke. Therefore, identifying the patients with high risk of thrombosis is crucial for advanced stroke warning. To better investigate the mechanism behind thrombus formation in the LAA, this study reconstructed the 3-D Left Atrium (LA) models of six AF volunteer patients by corresponding Computed Tomography (CT) images. Combine the advantages of Computational Fluid Dynamics (CFD), the blood flow field in LA both in AF and sinus heart rate states were studied. The risk of thrombus was evaluated based on the blood viscosity, shear rate thrombus prediction model and Time Average Wall Shear Stress (TAWSS), Oscillatory Shear Index (OSI), and Relative Residence Time (RRT) values. The results showed that the left atrium had lower blood flow velocity and TAWSS values at the LAA in both AF and sinus rhythm, thus the LAA is the most thrombogenic region in the LA. Besides, the RRT value of LAA was generally higher in AF than in sinus rhythm. Therefore, AF carries a higher risk of thrombosis.

## Introduction

Atrial fibrillation (AF) is the most common persistent arrhythmia (1). According to clinical data, the total prevalence of AF in adult (>18 years) in Shanghai was determined to be 0.88%, especially 6.7% for those over 80 years old (2). At the same time, the prevalence continues to rise with the aging of citizen population. The main risks of AF are complications, such as increased risk of stroke, heart failure, sudden death and cardiovascular disease, impaired cognitive function, and even dementia (3), among which stroke and systemic thromboembolism are the most common (4).

When AF occurs, cardiac output will decline subsequently (5). In certain corners of the atrium due to the loss of systolic function of the atria. As a result, blood flow becomes extremely slow, while the insoluble fibrin, platelets, and erythrocyte in the blood are more likely to clump together and form clots (6). The left atrial appendage (LAA) is such a corner, it is an accessory structure to the left atrium (LA) which is connected to the LA by a narrow channel (7) and contains a large number of internal folds making the surface unsmooth. The shapes of the LAA in patients with AF were classified into 4 morphological types, with “chicken wing” being the most common (48%), followed by “cactus” (30%), “windsock” (19%), and “cauliflower” (3%) (8). The above factors make it easier for thrombi to form and harder to be discharged, resulting in large scale thrombi over time. Once the thrombus in the LAA is dislodged, it will follow the blood flow to all parts of the body, causing ischemia and necrosis in many regions. The most common is dislodging into the intracranial arteries and blocking the cerebral vessels, causing a large cerebral infarction, which is named as stroke (9). Some studies indicate that the overall risk of stroke for patients with non-valvular AF is five times higher (10).

Stroke prevention is one of the priority tasks in the treatment of AF. Current treatments to reduce stroke risk include : (i) oral anticoagulation (11); (ii) LAA surgical exclusion (12); and (iii) percutaneous LAA occlusion (13). Their applicable conditions are different, and each of them has certain defects (11–13). Therefore, it is extremely important to choose the appropriate treatment according to the risk of stroke. The CHA_2_DS_2_-VASc score (14) is currently used clinically for AF stroke risk assessment. The score assesses stroke risk in patients based on clinical experience factors such as gender, age, hypertension and history of diabetes mellitus. The higher scores are associated with greater stroke risk. However, the risk of thrombus formation from blood flow inside the LAA was not taken into account (15, 16), which could lead to serious erroneous judgment.

The formation of thrombus inside the LA in AF is a specific fluid flow problem. Although the clinical studies can provide more intuitive data and results, they cannot reveal the mechanism of thrombosis caused by blood flow, which is difficult in both cardiology and biofluid mechanics. In recent years, with the rapid development of medical monitoring equipment and computer technology, a large number of scholars studied hemodynamic studies of LA/LAA by computational fluid dynamics (CFD).

Bosi et al. (17) simulated the hemodynamic behavior of four typical LAA morphologies in AF and normal conditions. The results indicated that thrombus is more likely to form in the LAA in AF and the morphology of the LAA affects the risk of thrombosis. Masci et al. (18) analyzed five different LAA models and found that the LAA with complex construction had low flow velocity and low vorticity, which led to a higher risk of thrombosis. Zhang and Gay (19) studied the effect of AF on internal flow in the LA and found that the LAA did not work in sinus rhythm and causes blood stagnation with thrombosis in AF. Wu et al. (20) analyzed the blood flow characteristics within different LAA models by numerical simulations, established the association between hemodynamic parameters and thrombosis, and provided a multi-parameter criterion for assessing the risk of thrombosis within the LAA. Wang et al. (21) explored the effect of AF on thrombosis by comparing blood flow in sinus rhythm with AF.

Chicken-wing type LAA, as one of the typical LAA types, is the main object in this paper. The mechanism of internal blood flow and thrombosis in this type of LAA under AF was studied combine the advantages of biofluid mechanics and the clinical diagnosis. For the numerical simulation, the LA model was obtained after inverse modeling of Computed Tomography (CT) images of six AF patients volunteers. The blood flow in LA was compared between sinus rhythm and AF, and the risk of thrombosis was predicted by thrombus prediction model to explore the hydrodynamic mechanism of thrombosis. It also provides reference data for further improving stroke risk stratification.

## Materials and methods

### Geometric models

**FIGURE 1:**
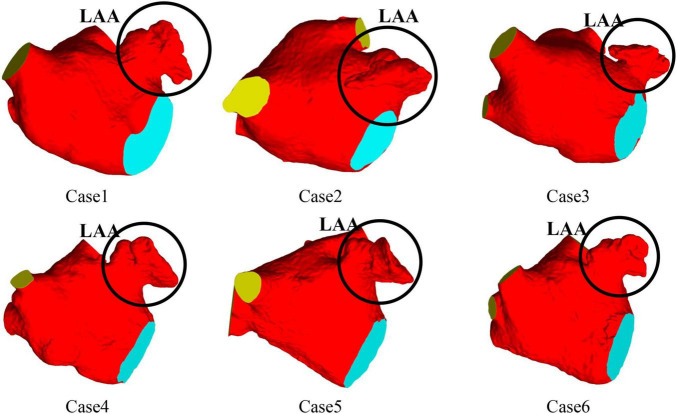
LA 3D models of six patients with AF.

CT images from six volunteers with AF and chicken wing shape LAA who underwent a scan at Xinhua Hospital, School of Medicine, Shanghai Jiao Tong University were used, with the permission from all volunteer patients. The 3D LA models were reconstructed based on the CT images of the patients, and the LA models shown in Figure 1, all of which were chicken-wing type. Table 1 shows the main geometric parameters of the patient’s LA. The mitral orifice area is the area of the flat section obtained by cutting the mitral valve orifice on the LA 3D model with a plane cutting tool. Among them, cases 2 and 3 had some degree of atrial enlargement. Case 6 showed five pulmonary vein openings which has one more pulmonary vein than normal. The study found that more than 10 % of patients with AF have additional pulmonary veins (22), which is a common anatomical variant. In addition, patients with additional pulmonary veins tend to have higher frequency of AF than those with other patterns (23). The subject of case 3 had stroke and none of the others had TIA or stroke.

**TABLE 1:** LA main geometric parameters of six patients with AF.

Case	LA volume (mL)	LAA volume (mL)	Mitral orifice area (cm^2^)	Number of pulmonary vein ports
1	136.08	19.33	9.57	4
2	193.22	15.70	7.60	4
3	213.40	20.98	9.69	4
4	84.03	21.97	6.20	4
5	106.69	25.01	7.32	4
6	122.69	19.48	8.70	5

### Thrombosis prediction model

Research shows that the non-Newtonian characteristics of blood cannot be neglected (24). Blood is a shear-dilution fluid that becomes less viscous as the shear rate increases. A large number of scholars have studied the rheological properties of blood and fitted the corresponding non-Newtonian model of blood (25), in which Quemada model can better reflect the rheological properties of blood (26) because of the consideration of Htc value, the hematocrit of blood. The range of Htc is 30 ∼ 60% (27), and the value of Htc in this work was 30%. In this paper, when the blood viscosity was higher than the blood viscosity threshold (0.00614 Pa⋅s) or the blood shear rate was lower than the shear rate threshold of 1 s^–1^, it was regarded as thrombosis (27). The mathematical expression is as bellow:


(1) $$\eta \,\left(\gamma \right)=\left\{ \begin{array}{cc} \eta_{p}\,{\left(1-\frac{K•H\,t\,c}{2}\right)}^{-2}, & \gamma \in \left\langle \gamma_{c},\infty \right\rangle \\ \eta_{c}, & \gamma \in \left\langle 0,\gamma_{c}\right\rangle \end{array}\right.$$

where η_P_ is the plasma viscosity (η_P_ = 0.001 Pa⋅s) (28), *K* is the inner viscosity of erythrocytes (Equation 2), *Htc* is the hematocrit (*Htc* = 30%), γ is the shear rate, η_*c*_ is the blood viscosity threshold (0.00614 Pa⋅s), and γ_*c*_ is the shear rate threshold (γ_*c*_ = 1 s^–1^).


(2) $$K=\frac{k_{0}+k_{\infty }\,{\left(\gamma \,/\,\gamma_{c}\right)}^{1\,/\,2}}{1+{\left(\gamma \,/\,\gamma_{c}\right)}^{1\,/\,2}}$$

where *K* is the inner viscosity of erythrocytes, *k*_0_ is the parameters characterizing blood behavior (*k*_0_ = 4.08) (29), *k*_∞_ is the parameters characterizing blood behavior (*k*_∞_ = 1.75) (29), γ is the shear rate, γ_*c*_ is the shear rate threshold (γ_*c*_ = 1 s^–1^).

In addition, it has been demonstrated that the formation of thrombus in the flow field is inextricably linked to the retention time of blood components (particles) in the vicinity of endothelial cells (30). Therefore, the risk of thrombus formation was reflected by the relative residence time (RRT) index in this paper. With the greater the value of RRT, the risk of thrombosis is increased.

RRT can be calculated from Oscillatory Shear Index (OSI) and Time Average Wall Shear Stress (TAWSS) (31). TAWSS reflects the biomechanical effect of Wall Shear Stress (WSS) at the LA wall. A smaller WSS value represents a lower flow velocity, which is associated with blood stasis and a higher risk of coagulation (32). OSI is a cause-free parameter whose value elucidates the deviation of WSS from the dominant direction of blood flow during a cardiac cycle (32). RRT combines WSS and OSI to reflect the residence time of blood particles near the wall. TAWSS, OSI and RRT are calculated as:


(3) $$T\,A\,W\,S\,S=\frac{1}{T}\,\int_{0}^{T}\left|W\,\vec{S}\,S\right|\,dt$$

(4) $$O\,S\,I=0.5\times \left(1-\frac{\frac{1}{T}\,\left|\int_{0}^{T}W\,\vec{S}\,S\,dt\right|}{T\,A\,W\,S\,S}\right)$$

(5) $$R\,R\,T=\frac{1}{\left(1-2\times O\,S\,I\right)\times T\,A\,W\,S\,S}$$

where *WSS* is the wall shear stress, *t* is the time, and *T* is the cardiac period.

### Solving process

Blood was considered to be a single-phase incompressible non-Newtonian fluid with a density of 1,060 kg/m^3^ in this work (33). Under isothermal circumstances, blood flow inside the LA was simulated for 10 cardiac cycles of 0.8 s each (37.5% atrial diastole and 62.5% atrial systole) with a time step of 0.005 s.

When AF occurs, the contractility of LAA and LA is reduced, especially in chronic AF, which causes rigidity on LA walls, preventing its proper contraction (34). So many scholars adopt the assumption of rigid wall in the CFD simulation of LAA in AF (17, 32, 35, 36). Some researchers indicated that the wall of LA set as rigid no-slip wall can simulate the worst case of AF, when there was almost no contraction of the LA (37). Studies have shown that fixed wall simulation can be reflect the flow patterns of LA and LAA well in dysfunctional atrium. In sinus rhythm, the risk of thrombosis can still be assessed, although with some limitations (38). Therefore, the heart wall in this paper was set up as a rigid no-slip wall.

**FIGURE 2:**
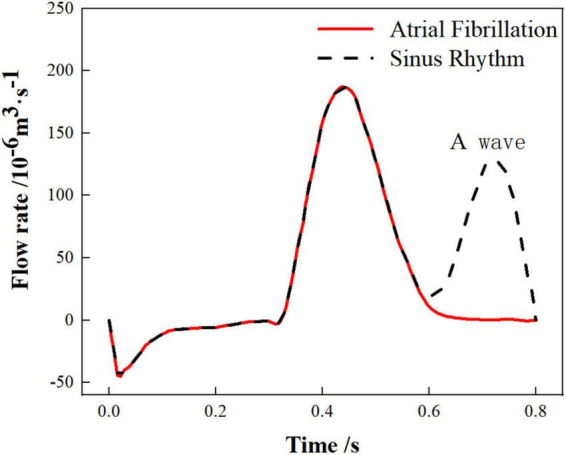
The variations in mitral flow within a single cardiac cycle.

The work in this paper only considers the effect of chicken wing shape on internal flow of LAA and influence of thrombosis risk in AF. Therefore, the pulmonary vein inlets in the 6 cases were all set as an open boundary condition with a constant pressure of 0 Pa and the flow rate of the mitral valve outlet were also the same. The flow rate across the mitral valve in the sinus rhythm adopted in the simulation was based on international regulation ISO5840-1:2015 (39) and the flow rate curve of a period (0.8 s) was shown as the black dashed line in Figure 2. For the simulation of AF, the flow rate across the mitral valve was obtained by removing the second atrial emptying wave (A wave) from the healthy mitral blood flow (40), shown as red line in Figure 2.

Although the flow rate curve at the mitral valve in all cases is the same, the periodic blood flow velocity boundary condition at outlet near mitral value were different due to the difference of the area.

Based on the mass conservation, the flow rate at the outlet surface near mitral valve in this simulation is the same as the flow rate at the real mitral valve. The area of outlet surface near mitral valve was measured with a measurement tool. Therefore, the velocity-outlet setting at the outlet surface near mitral valve in this simulation was acquired by the mitral blood flow rate divided by the area of outlet surface near mitral valve.

Piecewise polynomial function was used to fit the waveform of blood flow velocity, the one of blood flow velocity formula of mitral orifice in a cardiac period is obtained. Taking case 4 in sinus rhythm as an example, the expression of outlet velocity in 1 period is shown in Equation 6.


(6) $$v=\left\{ \begin{array}{cc} 13911\times t^{4}-3207.4\times t^{3}+261.02 & 0\leq t<0.08\\ \times t^{2}-7.8958\times t+0.0048 & \\ -63.807\times t^{4}+53.766\times t^{3}-16.404 & 0.08\leq t<0.33\\ \times t^{2}+2.2192\times t-0.125 & \\ 609.79\times t^{4}-1060.5\times t^{3}+662.82 & 0.33\leq t<0.6\\ \times t^{2}-174.95\times t+16.45 & \\ 1110.5\times t^{4}-3259.3\times t^{3}+3550.7 & 0.6\leq t<0.8\\ \times t^{2}-1702\times t+303.08 & \end{array}\right.$$

where *t* is time, *v* is the blood flow speed.

The CCL language in CFX is used to realize the above expression to be the periodic boundary condition of outlet. In the follow-up study, this method is used to fit the patients’ velocity waveforms at the outlet near the mitral value, which could also be used as the boundary condition of the calculation for personalized research. Laminar model was used for the calculation, which has been proved to be feasible (41).

In this paper, the additional variable RT (Residence Time) was modeled as a tracer passively transported with the flow and solved by the transport equation to reflect the residence time of blood in LA. The initial value of RT was set to 0, and the transport equation is (42):


(7) $$\frac{\partial R\,T}{\partial t}+v\cdot \nabla R\,T=D_{R\,T}\,\nabla^{2}R\,T+1$$

where: *t* is time, *v* is the blood flow speed, and *D*_*RT*_ is the self-diffusion rate of blood (*D_*RT*_* = 1.14 × 10^–11^ m^2^s^–1^) (42), and the source term “1” considers a unit increase in RT for each unit increase in time.

### Meshing and gird-independence verification

**FIGURE 3:**
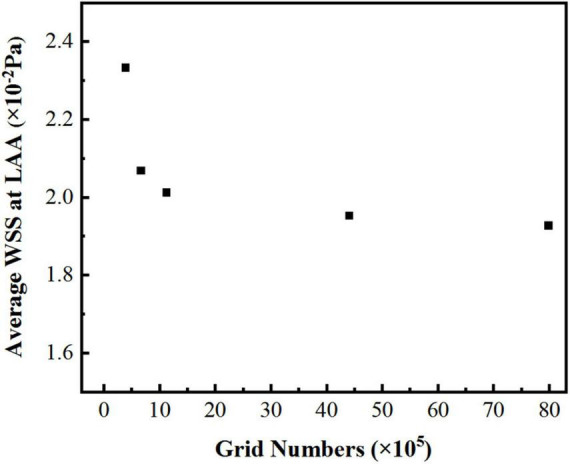
The average WSS values at LAA with different grid numbers.

In the simulation, the reconstructed solid model was divided with tetrahedral unstructured mesh. For each model of LA, five sets of meshes with varying mesh counts were split, and the average wall shear stress (WSS) at LAA was used to validate gird-independence, Figure 3 shows the mesh-independent result of one of the models. The average WSS at LAA tends to decrease as the number of grids increases. When the number of grids increased to 1,124,416, the increasing amplitude of average WSS was very weak as the grids continued to grow. Therefore, based on the consideration of calculation accuracy and reasonable allocation of computing resources, the grid with the grid number 1,124,416 was chosen for the calculation. Other models were validated using the same grid-independence procedure, and the appropriate grid was chosen for the computation.

## Results

### Flow distribution and residence time

**FIGURE 4:**
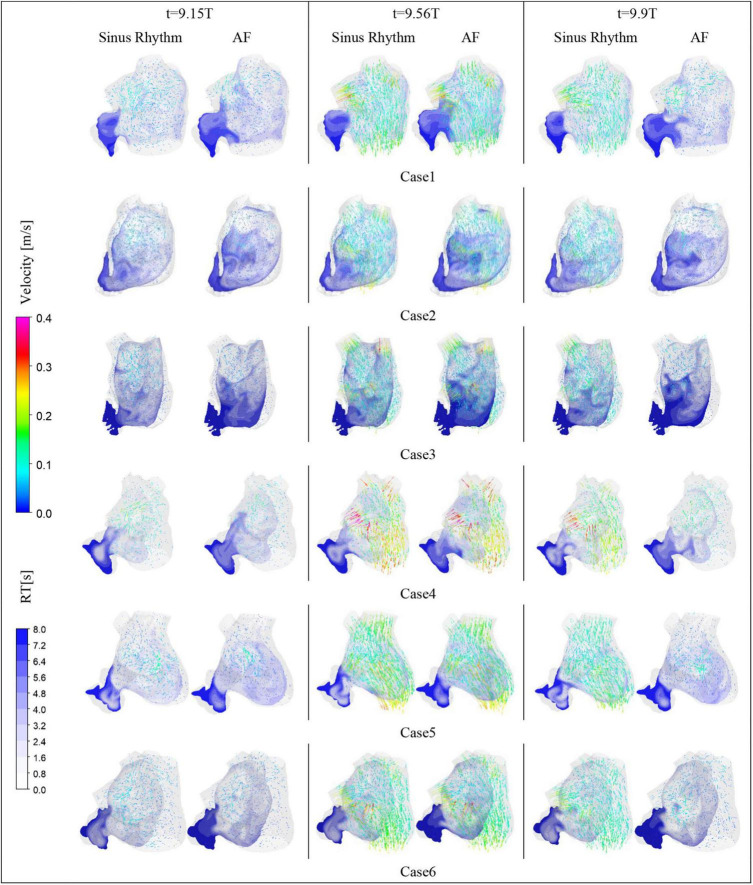
The blood flow velocity in LA and the distribution of residence time in a section.

**FIGURE 5:**
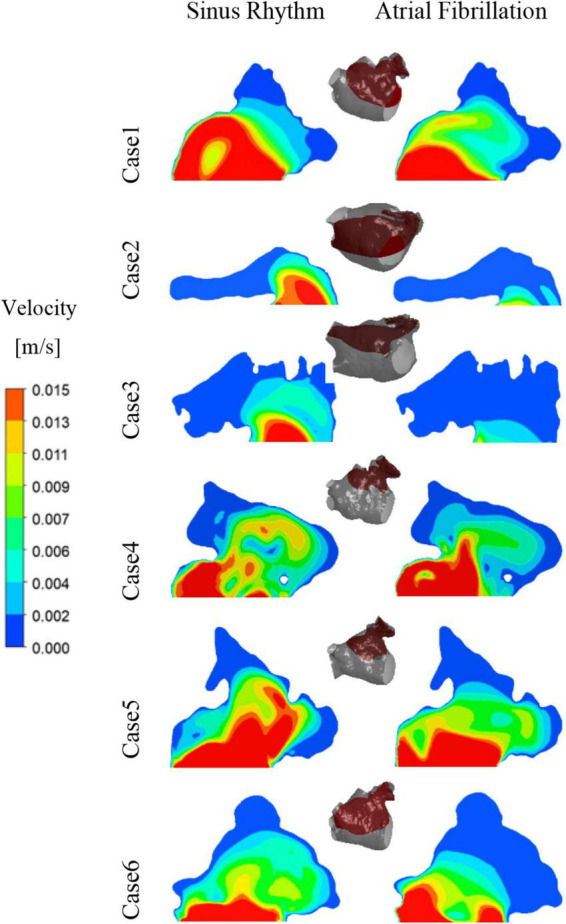
The velocity distribution of a section at LAA at *t* = 9.9T.

After convergence of the calculations, the data from the last cycle were selected for analysis and processing. T represents the period of one cardiac cycle (0.8 s) and t is the simulation time. Figure 4 shows the distribution of blood flow velocity inside the LA at three different time of *t* = 9.15T, *t* = 9.56T, and *t* = 9.9T in a normal sinus rhythm and AF. The distributions are practically both the same in the first half of the cardiac cycle, but in the latter period (*t* = 9.9T), the velocity inside the LA is much lower in the AF condition than in sinus rhythm. Simultaneously, the blood velocity in the LAA is much lower than elsewhere in the LA, both in sinus rhythm and AF. To clearly analyze the velocity distribution in the LAA, the velocity distribution on the cross section through the LAA was selected for analysis, as shown in Figure 5. In most part of the LAA, the velocity is only 0.015 m/s or less. Moreover, the flow velocity becomes lower at the regions further away from the LAA orifice. In AF state, the LAA has a lower blood velocity than in sinus rhythm, where blood flow is slower and more likely to cause thrombus formation.

RT value can indicate the residence time of blood components (particles) near endothelial cells, which is closely related to thrombosis (30). The risk of thrombosis was higher in regions with high RT values. The RT distribution over a certain cross-section through the LAA was analyzed. As shown in Figure 4, it can be seen that all cases have high residence time (above 5 cycles) at the LAA, and a greater range of high RT values in the AF state, especially in cases 2 and 3, which may be closely related to the swelling of the LA.

### Thrombosis prediction

Blood is a shear-dilution fluid that becomes less viscous as the shear rate increases. When the blood viscosity was higher than the blood viscosity threshold or the blood shear rate was lower than the shear rate threshold, it was regarded as thrombosis (27). In this work, Equation (1) was used to obtain the thrombus formation during the last cycle at sinus and AF heart rates in each case combined with the corresponding apparent viscosity and shear rate thresholds. TAWSS, OSI, and RRT values were also computed and solved at the LA to further validate the risk of thrombosis.

**FIGURE 6:**
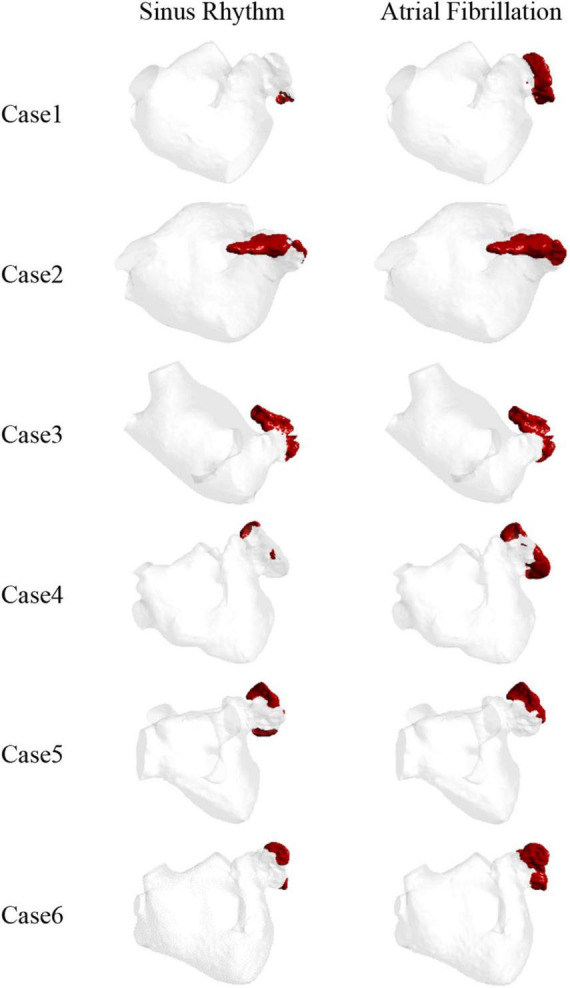
The thrombus distribution predicted by blood viscosity and shear rate.

The thrombus formation is shown in Figure 6. All the thrombus formation appeared at the LAA, and the extent of thrombus formation is greatly increased in the AF state. Therefore, AF may increase the risk of blood clots. Among the results, there is a significant thrombus formation in the LAA in sinus heart rate, while scarcely in case 1 and case 4. Meanwhile, compared to other cases there exists a greater extent of thrombosis both in AF and sinus heart rate in case 2 and case 3, which might be closely related to the enlargement of LA (43).

Therefore, the LAA is the most likely region for thrombosis in the LA. At the same time, thrombosis is more likely to form in AF than in sinus rhythm.

**FIGURE 7:**
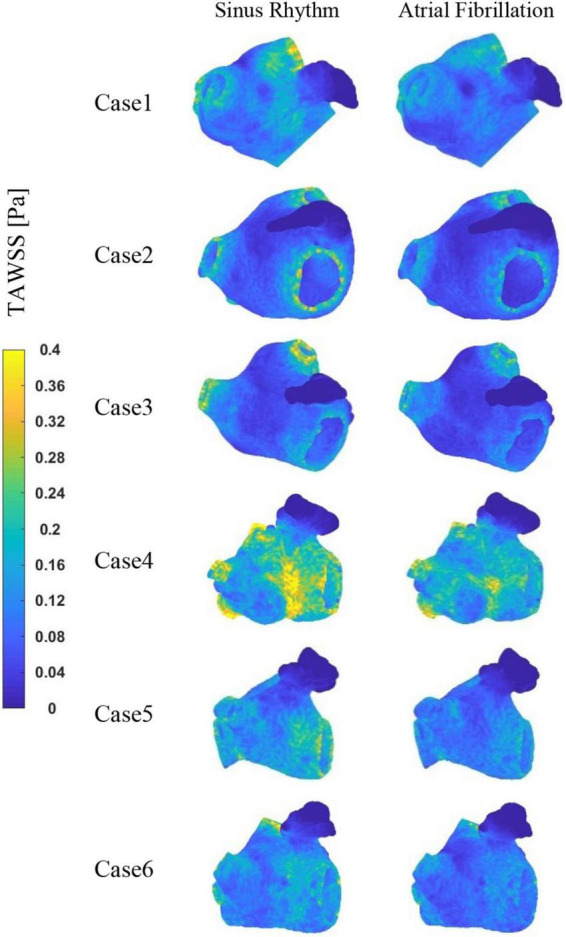
The distribution of TAWSS at LA.

The TAWSS values at the LAA are less than 0.04 Pa in each case, as shown in Figure 7, which is much lower than the TAWSS values at other sites inside the LA. While, the TAWSS values are higher at the pulmonary vein and mitral valve openings due to the increased flow velocity. Besides, as show in Table 2, the LA has lower TAWSS values and a higher risk of thrombosis in the AF condition. Furthermore, case 4 has a higher TAWSS values than the other examples due to the higher flow velocity.

**TABLE 2:** Mean TAWSS, mean RRT at LA and LAA and maximum RRT at LAA.

Case	Heart rate	LA (except LAA) average TAWSS (Pa)	LAA average TAWSS (Pa)	LA (except LAA) average RRT (Pa^–1^)	LAA average RRT (Pa^–1^)	LAA maximum RRT (10^4^Pa^–1^)
1	Sinus rhythm	0.13061	0.01898	18.43	673.68	1.44
	AF	0.10848	0.01675	28.83	2421.93	73.08
2	Sinus rhythm	0.09278	0.00764	36.43	89012.57	1078.11
	AF	0.07775	0.00301	54.99	161372.79	2250.46
3	Sinus rhythm	0.10147	0.01475	35.04	15763.56	302.30
	AF	0.08214	0.00939	72.55	186590.98	905.27
4	Sinus rhythm	0.22166	0.04037	11.33	237.18	0.77
	AF	0.18125	0.03137	15.36	351.36	2.14
5	Sinus rhythm	0.15117	0.01723	17.46	3548.55	139.17
	AF	0.12067	0.01717	24.50	5706.09	47.14
6	Sinus rhythm	0.13420	0.02992	18.19	4628.03	126.54
	AF	0.10697	0.01892	33.23	3055.47	5.08

**FIGURE 8:**
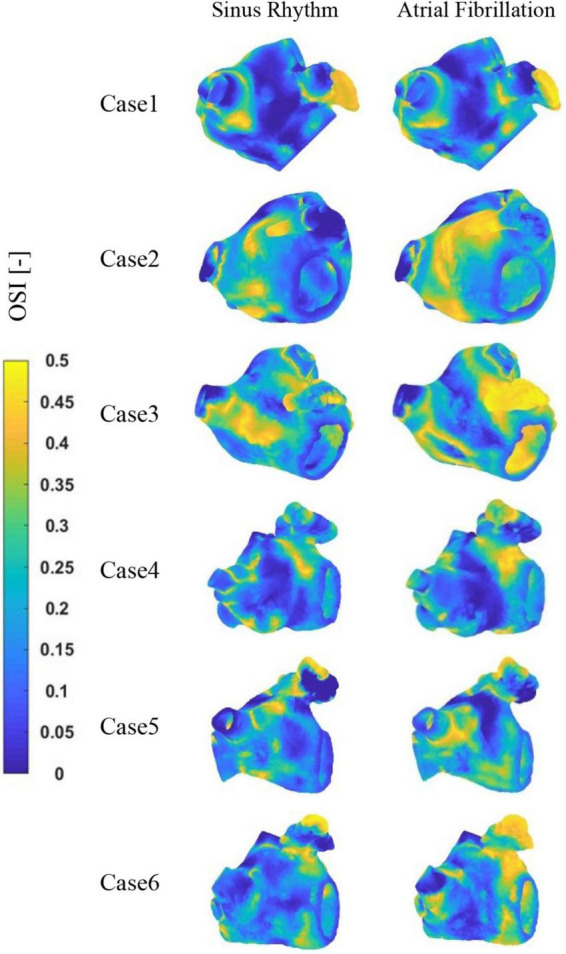
The distribution of OSI at LA.

The distribution of OSI at LA is shown in Figure 8. It can be observed that the high OSI values are mostly located in the LAA, the neck of the LAA orifice, and the neck of the pulmonary vein orifice. In addition, case 2 and 3 exhibit greater OSI values in other sections of the LA, which may be associated with cardiac hypertrophy of LA. Similarly, greater OSI values are associated with an increased risk of thrombosis in the AF state.

**FIGURE 9:**
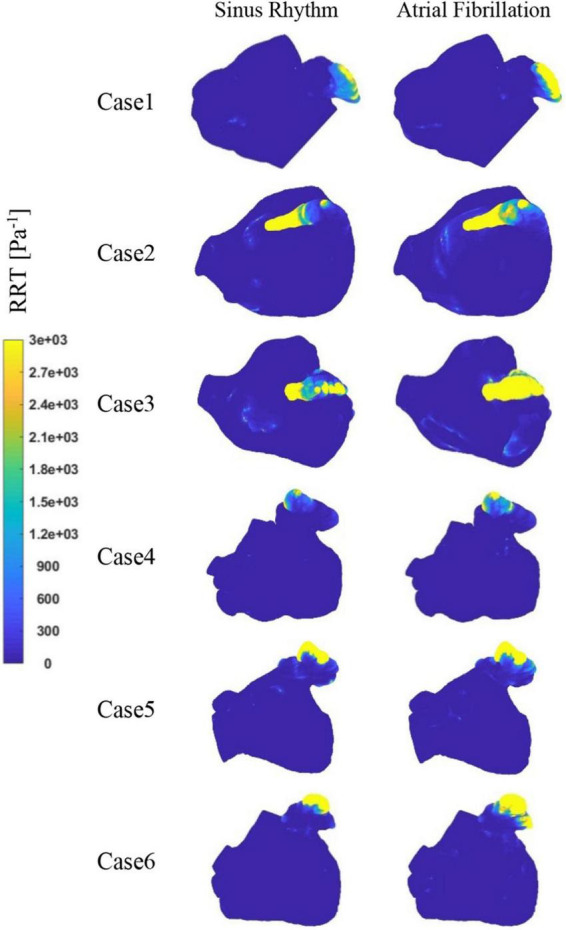
The distribution of RRT at LA.

Figure 9 depicts the distribution of RRT at LA. Combined with Table 2, it is evident that there are significantly higher RT values at the LAA than at other parts of LA. The difference of RRT values are more than two orders of magnitude, which demonstrates that the risk of thrombus formation at the LAA is much greater than at other parts of LA. Furthermore, there is a significant increase in RRT values inner LA in AF state. Table 2 shows that the mean RRT values inside the LAA in Case 6 are greater in sinus rhythm than in AF. Compared with the maximum RRT value, it has greater maximum RRT values in sinus rhythm than the AF, but this can only represent a minor fraction. In terms of the extent of the thrombotic risk region, there is a larger risk of thrombosis in the AF. Due to the existence of atrial enlargement, the RRT values also reveal that cases 2 and 3 have a greater risk of thrombosis than the other instances.

## Discussion

The incidence of AF and the related stroke is high in the world (44). LAA thrombosis, which is closely related to AF stroke, has become one of the hots and difficult points in clinical diagnosis and treatment of cardiology. The blood flow and thrombosis in LAA during the AF state is a special fluid flow problem, which is also a cross problem between cardiology and biofluid mechanics. With the continuous development and maturity of medical monitoring equipment and computer technology, more and more scholars combine the advantages of CFD (45) with the clinical diagnosis of LAA thrombosis, and use CFD method to study the LA blood flow information of specific patients to evaluate the risk of stroke (46). In this study, combined with the CT images of AF patients, the hydrodynamic model of LA is established to explore the hydrodynamic mechanism of thrombosis, and to evaluate the blood flow in LAA and the risk of thrombosis, in order to provide support for computer aided diagnosis of LAA thrombosis risk assessment. The results demonstrate that the LAA has lower blood flow velocity and TAWSS values than the LA in both AF and sinus rhythm, indicating that the LAA is the most thrombogenic region in the LA. Furthermore, the RRT value of LAA is often higher in AF than in sinus rhythm. As a result, AF contributes to raise the risk of thrombosis enormously. Furthermore, the cardiomegaly and additional pulmonary vein also have impacts on the internal blood flow field, which may increase the risk of thrombosis.

Through the analysis of flow field in six cases of different LA, it is found that the blood flow velocity in LAA is significantly lower than that in other parts of LA, and the low velocity of blood flow leads to the increase of blood residence time and the risk of thrombosis. This result is in agreement with that in reference (47). In addition, when AF occurs, the internal blood flow velocity of LAA is further reduced, and the risk of thrombosis is further increased, which is consistent with previous studies (17) and clinical results.

On this basis, the risk of thrombosis in LA via the thrombus prediction model and the RRT value based on wall shear force are further evaluated. The location of high risk of thrombosis is basically consistent with the distribution of high RRT value. From the prediction results of thrombosis, thrombosis is almost formed in LAA, and the degree of thrombosis is greatly increased in AF state. The distribution ranges of low TAWSS value and high RRT and OSI value are also wider. The study included two cases of enlarged heart, when LA has a certain swelling, the state of blood flows in LAA or even in the whole LA becomes worse. Once the blood flow rate is lower, the residence time and RRT value become higher, which increases the risk of thrombosis (43). However, in case 6 and case 5, it is found that the RRT of small regions in LAA exhibit higher values in sinus rhythm than AF, which also occurs in maximum RRT value unexpectedly. From the perspective of the distribution range of high RRT value, the range in AF state is significantly larger than that in sinus rhythm, thus the risk of thrombosis in AF is higher. This may be related to the rigid wall assumption, which needs further exploration.

## Limitations

Current work in this paper only considered the effect of chicken wing shape on internal flow of LAA and the influence on thrombosis risk in AF, therefore it still has some limitations such as the non-involvement of patient-specific boundary conditions. The velocity-outlet at outlet surface near mitral valve was set based on the international standard of ISO5840-1:2015 and the influence of the shape of the mitral value was not considered. The pressure-inlet at pulmonary veins was set as an open boundary condition with a constant pressure of 0 Pa. For the heart wall, a rigid no-slip wall was set up in the simulation.

However, the above limitations can be overcome step by step in subsequent studies. The pressure at pulmonary veins and the velocity at the mitral valve could be acquired by the clinical measurement. But the measurement on a surface more accurately is still a challenge. Dynamic CT data and dynamic mesh technology could be combined to describe the real wall motion of LA and mitral valve. But this implementation process is difficult at present.

Furthermore, only a qualitative analyze of TAWSS and RRT was done for evaluation of the risk of LAA thrombosis in this study. For the further quantitative analysis, more clinical data and existing experience (48, 49) are needed to support this work.

## Conclusion

In this work, a numerical simulation of Chicken-wing LAAs is carried out to study the thrombotic risk of this type in AF. Some flow field analysis such as blood velocity and residence time are used to evaluate the influence of AF on blood inside the LA. The risk of thrombus formation is evaluated by the blood viscosity and shear rate thrombus prediction model. By comparing and analyzing the numerical simulation results, the following conclusions are drawn.

(1) In both AF and sinus rhythm, the blood velocity inner the LAA is lower, and the blood stays for a longer time in the area which may cause higher thrombosis risk. This trend is also consistent with clinical results that the LAA is a high-risk region for thrombosis inside the LA.
(2) When AF occurs, the LAA has a smaller blood flow velocity which may cause a much greater residence time and RRT value, and a broader range of high RRT values. All these results increase the risk of thrombosis.
(3) When the patient’s LA become larger, the blood pooling at the LAA becomes more severe both in AF and sinus rhythm, and the risk of thrombosis is increased significantly.

These results demonstrate the high risk of thrombus formation within the LAA. Furthermore, AF greatly increases the risk of thrombus formation.

## Data availability statement

The raw data supporting the conclusions of this article will be made available by the authors, without undue reservation.

## Ethics statement

The studies involving human participants were reviewed and approved by the Ethics Committee of Xinhua Hospital Affiliated to Shanghai Jiao Tong University School of Medicine. Written informed consent for participation was not required for this study in accordance with the national legislation and the institutional requirements.

## Author contributions

JY, MC, JS, and XL conceived the study. JY, CS, MC, and JS made major contribution to the model construction and data analysis. HD, MC, and JS worked on CT image segmentation and provided the anatomical models. JY, CS, and XL drafted the manuscript. All authors contributed to data interpretation and critically revising.
